# Supplementary material for: Splice-Junction-Based Mapping of Alternative Isoforms in the Human Proteome
Source: Cell Rep. Author manuscript; Available in PMC 2020 Jan 15. (PMC6961840; doi:10.1016/j.celrep.2019.11.026)
Supplement: 2 [file NIHMS1546469-supplement-2.zip › DF1/PXD006675/Aorta/Aorta_19_NOL11_GLYSIEVLELR.pdf]

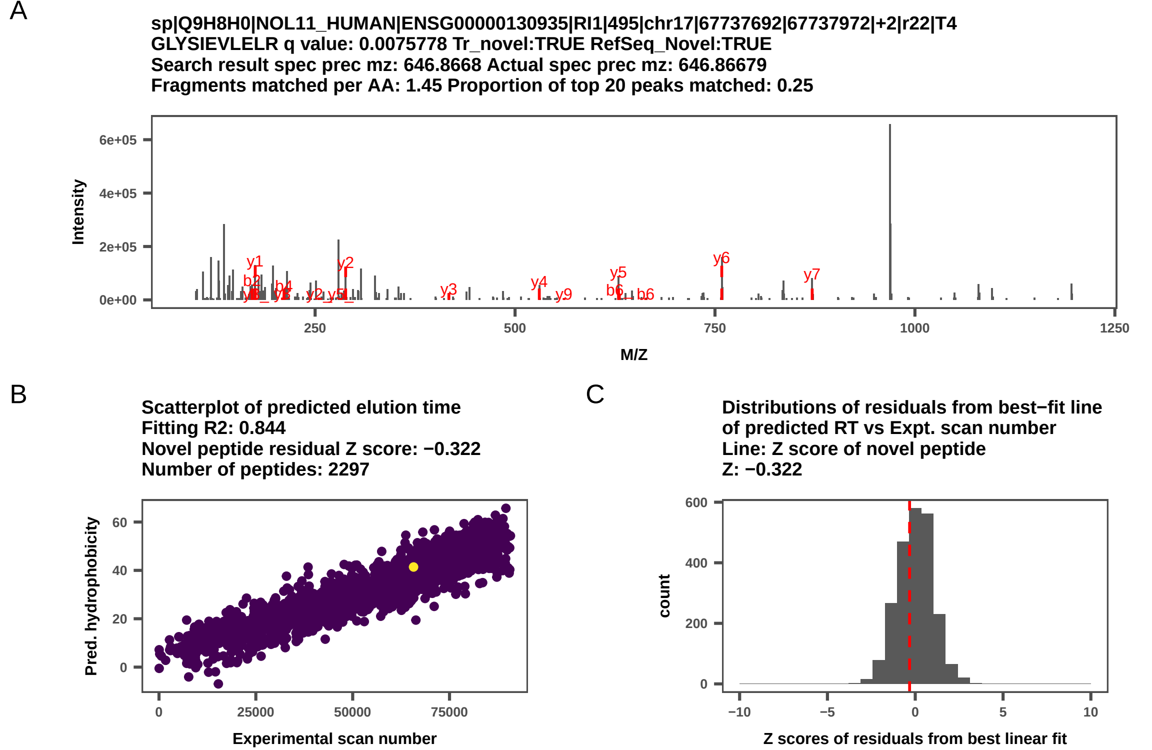
A
sp|Q9H8H0|NOL11_HUMAN|ENSG00000130935|RI1|495|chr17|67737692|67737972|+2|r22|T4
GLYSIEVLELR q value: 0.0075778 Tr_novel:TRUE RefSeq_Novel:TRUE
Search result spec prec mz: 646.8668 Actual spec prec mz: 646.86679
Fragments matched per AA: 1.45 Proportion of top 20 peaks matched: 0.25
Intensity
6e+05
4e+05
2e+05
0e+00
y1
b2
b4
y2
y3
y4
y9
y5
b6
b6
y6
y7
250
500
750
1000
1250
M/Z
B
Scatterplot of predicted elution time
Fitting R2: 0.844
Novel peptide residual Z score: −0.322
Number of peptides: 2297
Pred. hydrophobicity
60
40
20
0
0
25000
50000
75000
Experimental scan number
C
Distributions of residuals from best−fit line
of predicted RT vs Expt. scan number
Line: Z score of novel peptide
Z: −0.322
count
600
400
200
0
−10
−5
0
5
10
Z scores of residuals from best linear fit
